# The Great War: V. Sickness

**Published:** 1918-04-06

**Authors:** 


### THE GREAT WAR.

### V.—SICKNESS (*continued*).

### Immunity from Scurvy.

One of the most remarkable phenomena of the war on the Western Front is the absence of scurvy. Scurvy is a deficiency disease due to a want of accessory food factors or "vitamines." The Army in France has, comparatively, been living in the lap of luxury; our generous hosts have willingly shared their anti-scorbutic delicacies with our men, and to them our gratitude is due; but this comforting state of affairs may not persist under the pressure of world food shortage, and it is as well to take stock of our defences.

Anti-scurvy vitamine is present in all living tissues of plants, and to a much less degree in animals; it is absent from dried vegetables and seeds, being extremely susceptible to drying and comparatively easily destroyed by heat. Fresh-fruit juices, cabbage, and onions are specially rich in anti-scorbutic vitamine; potatoes and fresh meat are of less value, and milk is very poor in this respect.

Although dried seeds do not contain vitamine, this substance is restored when by exposure to moisture and heat they are allowed to germinate; in the easily obtainable dried peas, beans, or lentils we therefore possess an anti-scorbutic substitute for fresh vegetables when these are unobtainable. The pulses require to be soaked in water for twelve to twenty-four hours, according to the temperature, and then, after the water has been poured away, allowed to germinate for twelve to forty-eight hours, also according to the temperature (50° F. to 80° F.). They must be kept moist during this process and cooked as soon as germination is established. The discovery of this convenient anti-scorbutic is due to the researches of Miss H. Chick and Miss M. Hurne. It happens most opportunely, for the old-fashioned anti-scorbutic malt vinegar is no longer obtainable, and that which has taken its place is preserved in such a manner as to deprive it of its anti-scorbutic properties.

### War Nephritis.

Although a trivial source of wastage, war nephritis is full of etiological interest, and has provided material for constant research. It is a very curious complaint, in the causation of which various factors have been suggested, only to be excluded. It is peculiar to the white soldier actually serving in the trenches; officers very seldom suffer, and Indian soldiers and civilians in the war zone escape. Intense dyspnœa is a special feature, and this is associated with a morbid change in the lung, resembling the lesion due to inhalation of an irritant gas.

In the kidneys there is a characteristic affection of the glomeruli of the nature of diffuse intracapillary inflammation. Shaw Dunn and McNee, after prolonged and careful research, arrived at the conclusion that the disease is due to the selective action of an unknown poison. It is possible that the corpse-saturated soil of the battle area is in some way responsible.

### Burns and "Ambrene."

The treatment of burns has been revolutionised. Early in the war a French surgeon introduced the use of paraffin under the name of "ambrene." Its success was so great that the French War Department devoted a large hospital at Issy, in the suburbs of Paris, to the treatment of burns by this method. In it failures under the older methods may be seen under treatment by it and the results studied. In our opinion the comparison was so markedly in favour of the new method that it would seem almost criminal that its adoption should not be universal, at least until something even better is discovered. The points in its favour are the immediate and complete soothing of pain, rapid healing without loss of tissue, and the absence of constitutional symptoms. The treatment was eventually taken up by our War Department, who, after prolonged experiments, produced an ambrene substitute which they named "Paraffin No. 7," the formula being:

| | % |
|---|---|
| Re-sublimed Beta-naphthol .. .. | 0·25 |
| Eucalyptus Oil .. .. .. .. .. | 2·0 |
| Olive Oil .. .. .. .. .. .. | 5·0 |
| Vaseline .. .. .. .. .. | 25·0 |
| Paraffinum Durum .. .. .. | 67·75 |

The burn is first of all washed with normal saline and then thoroughly dried with gauze. The paraffin is heated to 55° C., at which it becomes of a syrupy consistence and easy of application. A layer is painted over the burn, a thin layer of wool is placed over it, and a second layer of paraffin applied, this being dressed with a thick layer of wool. The paraffin dressing is changed every twenty-four hours at first, the intervals increasing as healing progresses. The efficacy of this treatment depends largely upon the mechanical effect of the paraffin; the damaged tissues are protected and kept at rest. The addition of antiseptics of many kinds to the paraffin have been tried; whether they are essential for success is doubtful. Acriflavine 2 per cent. is the latest, and probably the best. It is said to produce a healthier type of granulations. Scarlet red in 10-per-cent. aqueous solution applied before the paraffin undoubtedly stimulates sluggish healing.

### Spirochætal Jaundice.

Although the number of cases of this disease is not large, it is of interest, as it involves the rat, which carries the causative germ, *Spirochæta icterohæmorrhagiæ*, in its kidneys and urine. It is a febrile disease characterised by febrile, icteric, and convalescent stages, the two former lasting each a week, and the last, marked by anæmia, emaciation,

☛ Previous articles appeared on Feb. 9 and 23, March 9 and 23, p. 523.

THE GREAT WAR—(*continued*).

and the urinary excretion of spirochætes, up to four weeks. The trenches abound with rats, and the question of their disposal is a controversial one. One side argue that their value as scavengers more than compensates the danger of plague and an occasional case of spirochætal jaundice, while the other side preach extermination; in practice a little trapping, a little poisoning, and a little hunting with dogs relieves the monotony of shell dodging, but has no appreciable effect on the rat population. Orders for the protection of food from rats, moreover, are difficult of execution; the bread ration makes a good pillow, and the paper wrapping of cheese is regarded by the rat as a *hors d'œuvre*.

Infectious Diseases.

Owing to the strength of the defence against infectious diseases, it is difficult for any one of them to make much headway. Measles heads the list; it is difficult to control, but the type is mild and the loss of fighting strength not serious. Small outbreaks of diphtheria occasionally occur, but dissemination is prevented by the immediate segregation of the affected and the search for and sterilisation of carriers. Sporadic cases of cerebrospinal meningitis occur from time to time; commensal infection is most rare, and change of area with relief of overcrowding are usually sufficient safeguards.

(*To be continued.*)

